# MiR-200c Increases the Radiosensitivity of Non-Small-Cell Lung Cancer Cell Line A549 by Targeting VEGF-VEGFR2 Pathway

**DOI:** 10.1371/journal.pone.0078344

**Published:** 2013-10-30

**Authors:** Liangliang Shi, Sheng Zhang, Hongge Wu, Lilin Zhang, Xiaofang Dai, Jianli Hu, Jun Xue, Tao Liu, Yichen Liang, Gang Wu

**Affiliations:** Cancer Center, Union Hospital, Tongji Medical College, Huazhong University of Science and Technology, Wuhan, China; The University of Tennessee Health Science Center, United States of America

## Abstract

MicroRNAs (miRNAs) have been demonstrated to participate in many important cellular processes including radiosensitization. VEGF family, an important regulator of angiogenesis, also plays a crucial role in the regulation of cancer cell radiosensitivity. VEGFR2 mediates the major growth and permeability actions of VEGF in a paracrine/autocrine manner. MiR-200c, at the nexus of epithelial-mesenchymal transition (EMT), is predicted to target VEGFR2. The purpose of this study is to test the hypothesis that regulation of VEGFR2 pathway by miR-200c could modulate the radiosensitivity of cancer cells. Bioinformatic analysis, luciferase reporter assays and biochemical assays were carried out to validate VEGFR2 as a direct target of miR-200c. The radiosensitizing effects of miR-200c on A549 cells were determined by clonogenic assays. The downstream regulating mechanism of miR-200c was explored with western blotting assays, FCM, tube formation assays and migration assays. We identified VEGFR2 as a novel target of miR-200c. The ectopic miR-200c increased the radiosensitivity of A549 while miR-200c down-regulation decreased it. Besides, we proved that miR-200c radiosensitized A549 cells by targeting VEGF-VEGFR2 pathway specifically, thus leading to inhibition of its downstream pro-survival signaling transduction and angiogenesis, and serves as a potential target for radiosensitizition research.

## Introduction

Patients suffered from non-small-cell lung cancer (NSCLC) account for approximately 85% of all lung cancer cases [1], [2]. Radiotherapy (RT) is a powerful modality widely used in clinic against cancer cells. However, many of them exhibit intrinsic or acquired radioresistance to RT leading to treatment failure [3]. Accumulating evidence shows that radioresistance is not only by intrinsic characteristics but a result of interactions between cancer cells and microenvironment factors. The paracrine/autocrine role of vascular endothelial growth factor (VEGF) by binding to its receptors is one important component of tumor microenvironment and its self regulation. Suppression of VEGF gene expression could enhance the radiosensitivity of cancer cells [4], [5]. And VEGFR2 is usually considered to mediate the main function attributed to VEGF. Radiation therapy combined with VEGFR2 and EGFR blockade caused a significant enhancement of antitumor effects in an orthotopic model of lung cancer [6]. Molecular inhibition of VEGFR2 could enhance tumor radiation response through molecular targeting of tumor vasculature [7]. So paracrine signaling from host VEGF to cancer cell VEGFR2 might be a significant component of RT failures [8].

MicroRNAs (miRNAs) are a group of small non-coding RNAs which suppress their target expression by binding to the 3′ untranslated region (3′UTR). One study that identified rat lung-specific miRNAs by miRNA microarray revealed that miR-200c expressed specifically in normal rat lung tissues [9]. And loss of miR-200c expression could induce an aggressive, invasive and chemoresistant phenotype in non-small-cell lung cancer [10]. Besides, independent studies showed that restoration of miR-200c could increase the sensitivity to chemotherapy agents in various tumors [11], [12]. So does miR-200c play a similar role in radiotherapy of non-small-cell lung cancer?

Bioinformatic analysis showed that VEGFR2 was a good predicted target of miR-200c with two binding sites. In this experiment, we investigated whether VEGFR2 could be regulated by miR-200c, leading to modulation of the radiosentivitiy of A549 cells.

## Results

### VEGFR2 is a Direct Target of miR-200c

**Figure 1:**
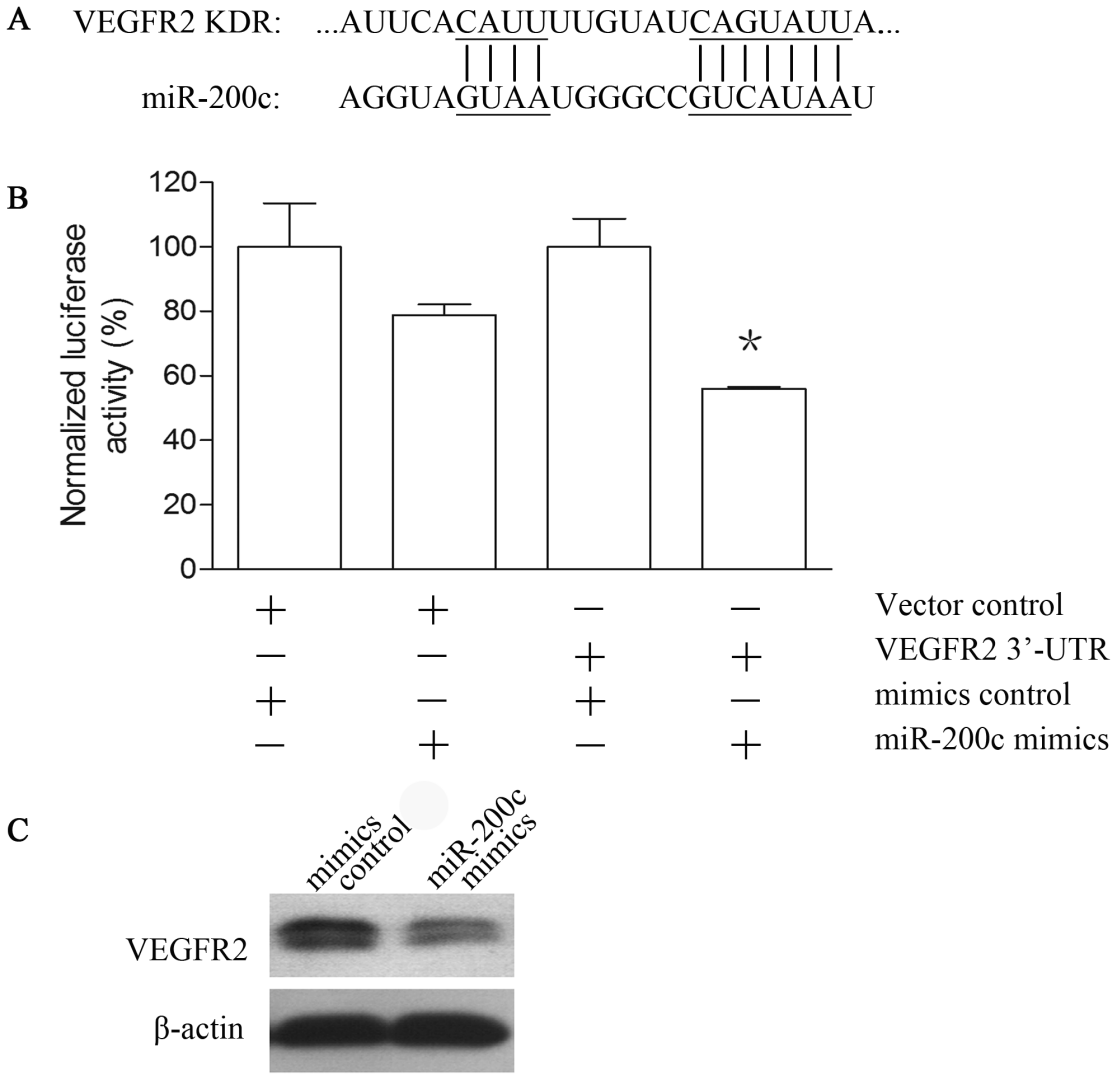
VEGFR2 is a direct target of miR-200c. (A) miR-200c target site residues at 3′-UTR of gene VEGFR2 inspected by bioinformatics. (B) The pLuc-VEGFR2–3′UTR construct contains a wild-type sequence of the 3′UTR of VEGFR2. The pLuc-VEGFR2–3′UTR construct was co-transfected with miR-200c mimics into A549 cells. Luciferase activity was detected 48 h after transfection. And the ratio of normalized luciferase value is shown. (C) VEGFR2 protein expression in A549 cells were measured by western blot 48 h post-transfection. Each experiment was repeated three times. Standard errors of the mean are shown by error bars. *indicates p<0.05 compared to the control.

Bioinformatic analysis revealed that VEGFR2 (vascular endothelial growth factor receptor 2) is a predicted target of miR-200c which may directly inhibit its gene expression (Fig. 1A). A549 cells were transfected with miR-200c mimics (50 nM) or miR-200c inhibitors (100 nM) to increase or decrease miR-200c expression. Mimics controls (50 nM) or inhibitors controls (100 nM) were transfected into A549 cells as negative controls respectively. Realtime PCR showed that miR-200c mimics and miR-200c inhibitors could significantly increase or decrease miR-200c expression of A549 (Data not shown). To further confirm whether miR-200c could directly bind to 3′UTR of VEGFR2, we carried out dual luciferase reporter gene assay using pLuc-VEGFR2–3′UTR plasmid in A549 cells. Transient transfection of A549 cells with pLuc-VEGFR2–3′UTR plasmid and miR-200c mimics led to a significant decrease of luciferase activity as compared to the controls (Fig. 1B). To examine if miR-200c could affect VEGFR2 protein expression in A549 cells, we carried out western bolt assays and found that miR-200c mimics reduced the protein expression of A549 significantly compared to the controls (Fig. 1C).

### MiR-200c Mimics Increased the Radiosensitivity of A549 Cells while miR-200c Inhibitors Decreased It

**Figure 2:**
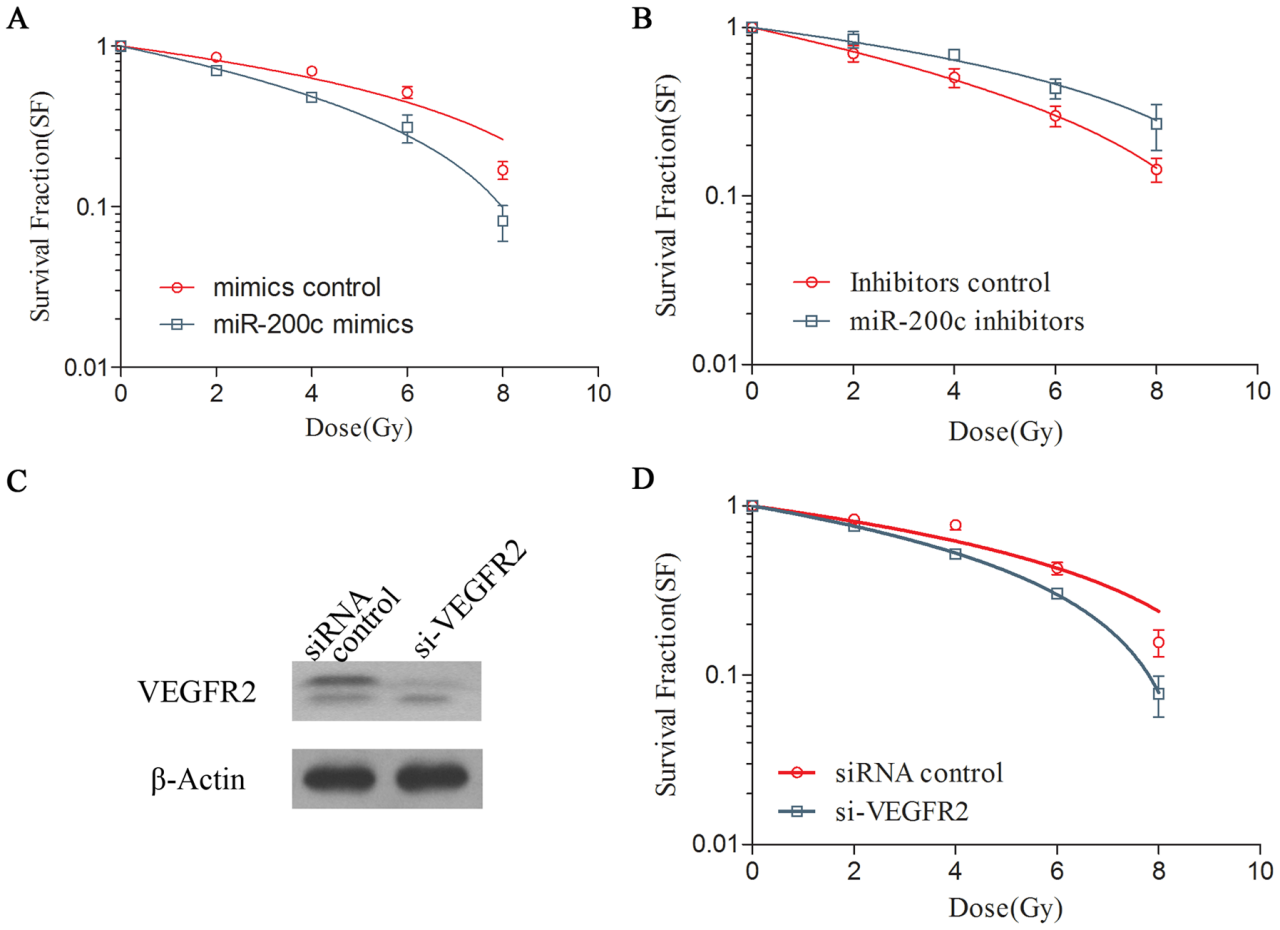
Ectopic expression of miR-200c increased the radiosensitivity of A549 cells by inhibiting VEGFR2 expression. Overexpression of miR-200c (A) enhanced the radiosensitivity of A549 cells to IR treatment, while miR-200c inhibition (B) showed the opposite effect. The si-VEGFR2 construct inhibited VEGFR2 protein expression 48 h post-transfection (C) resulting in the radiosensitization of A549 cells (D). Mimics control, inhibitors control and siRNA control were transfected into A549 cells respectively as negative control. The clonogenic survival assays were repeated three times with similar results. Standard errors of the mean are shown by error bars.

To study whether miR-200c affected cell radiosensitivity, A549 cells were transfected with miR-200c mimics or inhibitors. Colony survival assays, a gold standard for radiosensitivity estimation, were assessed following 0–8 Gy radiation. Transfection of A549 cells with miR-200c mimics (50 nM) significantly decreased cell survival after radiation exposure (SER_0.5_ 1.47, Fig. 2A). Conversely, transfection of A549 cells with miR-200c inhibitors (100 nM) caused a significant increase of cell survival following 0–8 Gy radiation compared to the controls (SER_0.5_ 0.79, Fig. 2B).

VEGFR2 knockdown was done by RNA interference using lipo2000 transfection reagent. Colony survival assay showed the knockdown of VEGFR2 protein expression levels (Fig. 2C) resulted in an increase in the radiosensitivity of A549 (SER_0.5_ 1.34) (Fig. 2D).

### VEGF Neutralization Abolished the Radiosensitization Effect of miR-200c

**Figure 3:**
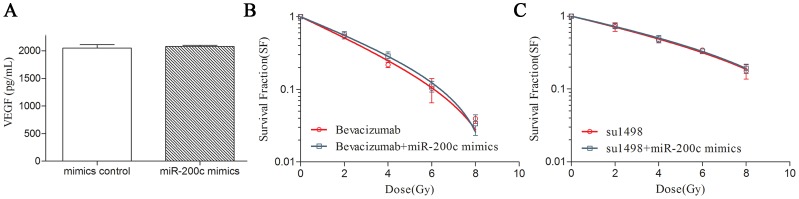
Blocking of VEGF-VEGFR2 pathway abolished the radiosensitization effect of miR-200c. (A) Elisa assay was used to detect VEGF secretion changes after transfection of miR-200c mimics into A549 cells. Bevacizumab (B) and su1498 (C) were applied to block VEGF-VEGFR2 pathway. Then clonogenic assays were carried out to examine the radiosensitization effect of miR-200c after VEGF-VEGFR2 blockage. Each experiment was repeated three times. Standard errors of the mean are shown by error bars.

We examined the variation of VEGF secreation in the extracellular medium after ectopic miR-200c expression, using VEGF elisa assay. And no significant changes of VEGF secreation following miR-200c intervention have been proved after duplicate tests (Fig. 3A). Bevacizumab (Bev, 1 mg/ml), a VEGF-specific blocking monoclony antibody, was then added to the culture medium of A549 cells to decrease extracellular VEGF concentration 1 hour before transfection. Colony survival assays were carried out to investigate the radiosensitization effect of miR-200c with or without VEGF neutralization. The results showed that miR-200c couldn’t decrease post-radiation survival fraction without VEGF-induced activation (Fig. 3B).

### VEGFR2 Blockage Abolished the Radiosensitization Effect of miR-200c

VEGFR2 was blocked by su1498, a tyrosine kinase inhibitor of vascular endothelial growth factor receptor 2 (VEGFR2). Su1498 was added into the growth medium of A549 cells to a final concerntration of 5 uM one hour before transfection. Clonogenic assays were carried out to access the survival of A549 cells after different doses of radiation. The results showed that rdiosensitization by VEGFR2 inhibition was significantly blocked by inhibiting VEGFR2 pathway (Fig. 3C). So miR-200c increased the radiosensitivity of A549 mainly by targeting VEGFR2 pathway.

### MiR-200c Inhibited the Activation of ERK1/2 and Akt

**Figure 4:**
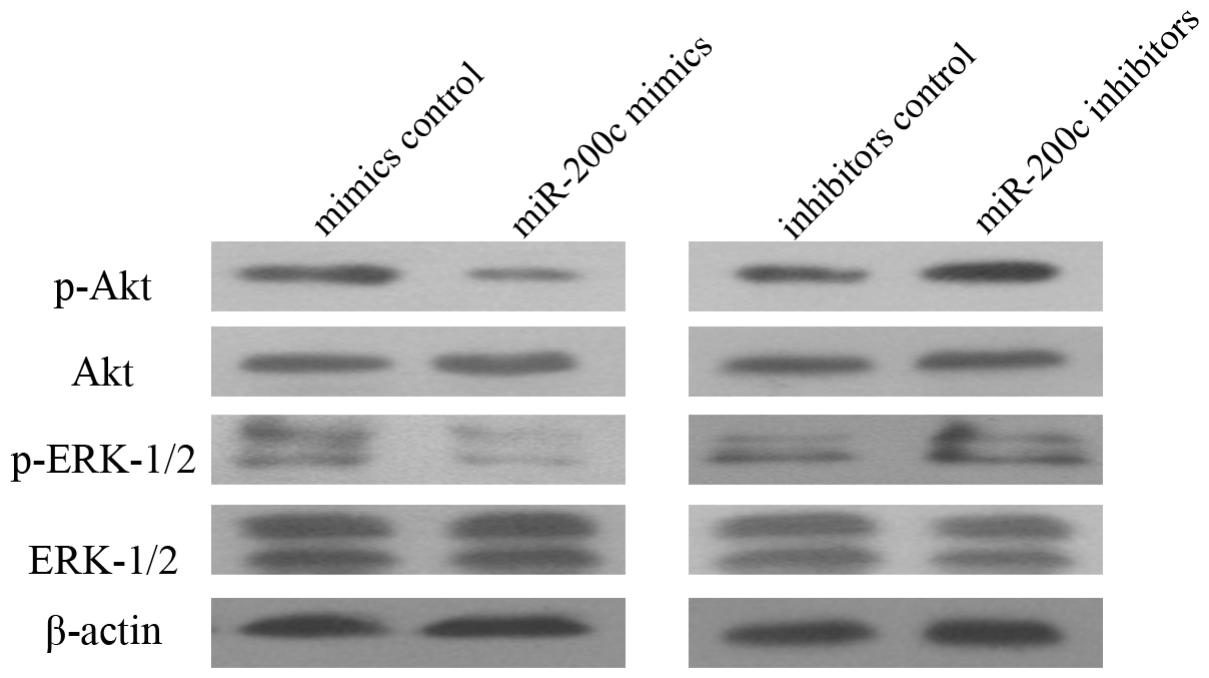
Western blot analysis of VEGFR2 downstream signaling transduction. Ectopic expression of miR-200c inhibited phosphorylation of Akt and ERK1/2, inhibition of miR-200c increased activation of Akt and ERK1/2 pathway. Each experiment was repeated three times.

To further study whether the downstream signal transduction pathways of VEGFR2 were involved in radiosensitization of A549, we investigated MAPK and AKT pathways that were most important downstream pro-survival pathways of VEGFR2. A549 cells were transfected with miR-200c mimics or inhibitors. Then we examined the expression level and activation of ERK1/2 and Akt which are the effecter molecules of MAPK and AKT pathway. Compared to cells treated with mimics controls, the activation of ERK1/2 and Akt indicated by p-ERK1/2 and p-Akt were significantly down-regulated in A549 cells treated with miR-200c mimics (Fig. 4). On the contrary, miR-200c inhibitors increased activation of ERK1/2 and Akt compared to inhibitor controls. These data suggest that activation of ERK1/2 and Akt modulate the radiosensitization effect of miR-200c (Fig. 4).

### MiR-200c Inhibited Angiogenesis and Migration of HUVEC Cells

**Figure 5:**
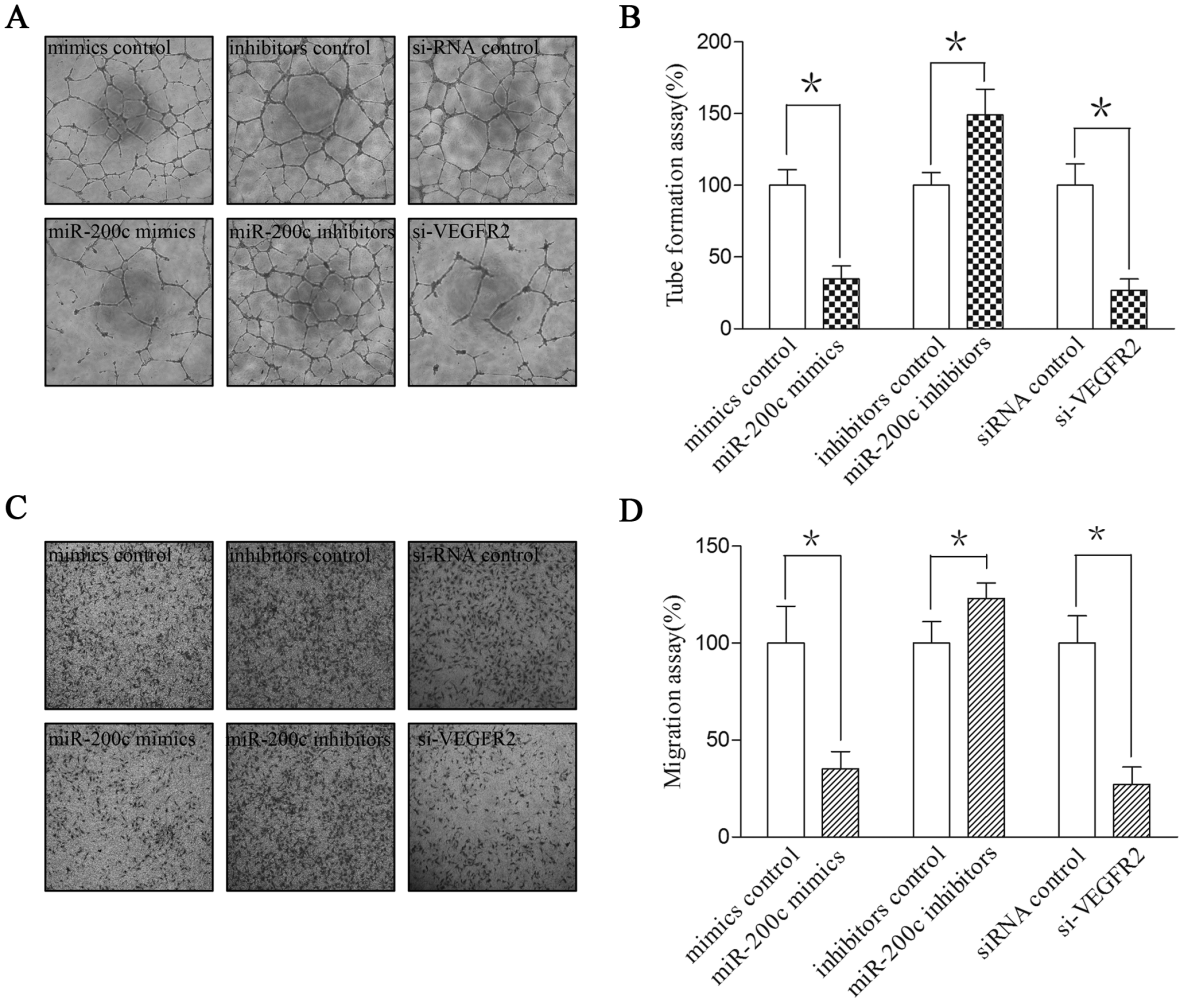
miR-200c inhibited angiogenesis of endothelial cells. HUVECs were transfected with miR-200c mimics, miR-200c inhibitors or si-VEGFR2. (A) HUVECs were cultured on Matrigel 48 h after transfection. Representative images of capillary-like structures formed and (B) the length of capillary structure were quantified. (C) The transfected HUVECs were also seeded onto the upper chamber of 8.0-mm pore size 24-well transwell plates to investigate cell migration ability. (D) The migratory cells were counted and statistically analyzed. Each experiment was repeated three times. Standard errors of the mean are shown by error bars. *indicates p<0.05 compared to the control.

We further investigated that whether miR-200c, a direct regulator of VEGFR2, could regulate angiogenesis of endothelial cells. 48 hours after transfection, HUVEC cells were starved overnight and seeded in 96 well plates that were pre-coated with Matrigel. After incubation for 16 hours, miR-200c-transfected cells showed a significant impairment of tube formation ability. Besides, down-regulation of VEGFR2 by si-VEGFR2 could also inhibit the angiogenesis of HUVEC cells. Since migration is an important step of angiogenesis, we also detected the impact of miR-200c on migration of HUVEC cells. As shown in Figure 5, ectopic expression of miR-200c significantly inhibited angiogenesis and migration of HUVEC cells. On the other side, suppression of miR-200c increased tube formation and migration by about 30% for HUVEC cells.

## Discussion

Previous work from our group and others has suggested that VEGF modulates tumor survival through a variety of ways including radiosensitization [13]–[15]. VEGF induces biological effects in a paracrine/autocrine manner by binding to its receptors, especially VEGFR2 (KDR, FLK-1). Some preclinical studies have shown that therapeutic benefits of radiotherapy could be enhanced when combined with inhibitors of VEGFR2 [16]. Targeting VEGFR2 pathway provides a new way to overcome radioresistance in the radiation therapy of various cancers. Recently, some miRNAs were demonstrated to modulate the radiation therapy of cancer cells. MiR-200c is the key regulator of epithelial-to-mesenchymal transition (EMT) which is associated with embryogenesis, cancer progression and metastasis. And miR-200c is expressed specifically in normal rat lung tissues [9] while loss of miR-200c expression induced an aggressive, invasive and chemoresistant phenotype in non-small cell lung cancer [10]. VEGFR2 is a predicted target of miR-200c by bioinformatic analysis (http://www.targetscan.org). So it’s important to find out whether miR-200c could modulate the radiosensitivity of cancer cells by targeting VEGFR2 pathway.

By dual luciferase reporter gene assays and western blot analysis, we proved VEGFR2 as a direct target of miR-200c. Then we tested the effects of ectopic expression of miR-200c on the radiosensitivity of A549 cells. We found that up-regulation of miR-200c with miR-200c mimics significantly reduced VEGFR2 protein expression and increased the radiosensitivity of A549 cells after different doses of radiation. However, down-regulation of miR-200c expression with miR-200c inhibitors increased cell survival and decreased radiosensitivity. We further constructed si-VEGFR2 to inhibit VEGFR2 protein of A549 cells. The results showed that decreasing VEGFR2 expression using si-VEGFR2 could enhance radiosensitivity in A549 cells, which was consistant with the effects of VEGFR2 down-regulation in other tumor cell lines [17], [18]. So miR-200c could enhance radiosensitivity of A549 by inhibiting VEGFR2 expression.

However, one single miRNA could regulate hundreds of target genes. So does miR-200c radiosensitize A549 cells mainly through targeting VEGFR2 pathway? Su1498 is a tyrosine kinase inhibitor specific for VEGFR2. We found that miR-200c couldn’t radiosensitize A549 while VEGFR2 was inhibited by su1498. Since VEGF is the main ligand of VEGFR2 functioned in a paracrine/autocrine manner, we first examined variation of VEGF secretion after miR-200c transfection and found that miR-200c mimics couldn’t affect VEGF secretion of A549. Then we neutralized secreted VEGF outside A549 cells with bevacizumab before transfection of miR-200c, and got a negative result just like VEGFR2 inhibition. So this suggests that miR-200c could radiosensitize A549 cells by targeting VEGF-VEGFR2 pathway.

Then we further investigated downstream mechanisms of miR-200c radiosensitization. MAPK and PI3K/AKT signaling transduction pathways are two important downstream signaling pathways of VEGFR2 [19]. Direct targeting and inhibition of these two pathways may increase radiosensitivity of cancer cells. Phosphorylation of p44/42-MAPK (Thr202/Tyr204) and Akt (Ser473), which are key molecules of MAPK and PI3K/AKT signaling pathways, usually increases survival of cancer cells after radiation. In our study, western blot assays were taken to examine the phosphorylation level changes of Akt and ERK1/2 (p44/42-MAPK) after tansfection of miR-200c mimics or inhibitors into A549 cells. We found that whereas increased expression of miR-200c led to an inhibition of Akt and ERK1/2 phosphorylation, decreased expression of miR-200c enhanced Akt and ERK1/2 phosphorylation in A549.

Hypoxic microenvironment extrinsic to cancer cells contributes significantly to radioresistance of radiotherapy [7]. Suppression of angiogenesis significantly enhanced radiosensitivity of cancer cells. And VEGFR2, a key mediator of angiogenesis, could affect the tumor microenviroment (TME) that modulate cancer cell radiosensitivity. Our data suggest that ectopic expression of miR-200c suppressed angiogenesis of HUVEC cells.

Besides, we also investigated other mechanisms of miR-200c-VEGFR2 interaction. With FCM or MTT assays, we found no significant effects of miR-200c on cell cycle, apoptosis or proliferation of A549 cells (Data not shown).

Taken together, our results indicate that miR-200c-mediated inhibition of the VEGF-VEGFR2 signaling cascade could radiosensitize human NSCLC cell line A549 cells to radiation, which is a therapeutic target of radiosensitization.

### Conclusions

Our data suggest that miR-200c could significantly radiosensitize A549 cells by targeting VEGF-VEGFR2 pathway.

## Materials and Methods

### Cell Culture

A549 cells, obtained from the Cell Bank of Chinese Academy of Sciences (Shanghai, China), were cultured in RPMI 1640 medium containing 10% fetal bovine serum at 37°C in a humidified 5% CO_2_ atmosphere. HUVEC cells from the American Type Culture Collection (ATCC) were cultured in endothelial cell medium (ECM; Sciencell, USA). The culture medium was renewed every 2–3 days. And the cells were passaged at 1∶6 every 4 days following trypsinization with 0.05% trypsin-EDTA.

### Transient Transfection and Cell Treatments

MiR-200c mimics, miR-200c inhibitors, si-VEGFR2, mimics controls, inhibitors controls, and siRNA controls were synthesized by Ribobio (Guangzhou, PR China). Cells were seeded at 4×10^5^ per cell into 6-well plates one day before transfection. Lipofectamine 2000 (Invitrogen) transfection reagent was employed to transfect cells with miR-200c mimics (50 nM), miR-200c inhibitors (100 nM) or VEGFR2 small interfering (si) RNA (100 nM). No-targeting mimics controls (50 nM), inhibitors controls (100 nM) or siRNA controls (100 nM) were transfected into cells as negative controls, respectively. 6 hours after transfection, cell growth medium was removed and incubated in media containing 5% FBS for another 24–72 hours. Further experiments were carried out with transfected cells or cell lysis. Su1498 (Santa Cruz Biotechnology) or Bevacizumab (Roche) was administrated 60 minutes before transfection.

### Identification of miR-200c Targets

Using Lipofectamine 2000 (Invitrogen), A549 cells were transfected with luciferase reporter constructs containing the 3′UTR of VEGFR2 or the negative control vectors (Promega). The transfection mixtures contained 100 ng of fireflyluciferase reporter plasmid and 50 nM of synthetic miR-200c duplex. A549 cells were collected 48 hours after transfection, and luciferase activity was measured using the Dual Luciferase Reporter Assay system (Promega).

### Clonogenic Assay

Sensitivity of A549 cells to irradiation was determined by clonogenic assay after the variable doses of radiation (0Gy, 2Gy, 4Gy, 6Gy, 8Gy) using a linear accelerator (Primus K, Siemens, Munich, Bayern, Germany). After incubation for 10–14 days, colonies formed were fixed with methanol and stained with 1% crystal violet. Only colonies consisting of more than 50 cells were counted. The data were fitted to linear-quadratic model using Sigmaplot software, where survival curves were generated and radiosensitivity parameters were calculated. Experiments were repeated three times.

### Measurement of VEGF Level

The level of VEGF secreation by A549 cells was evaluated by human VEGF-ELISA kit (R&D, USA) according to the manufacturer’s protocol.

### Western Blotting

Cells lysis was prepared and separated by 8–12% SDS-PAGE, followed by transferring onto PVDF membranes (Millipore). For detection, membranes were incubated with specific primary antibodies and secondary antibodies sequentially. Primary antibodies against VEGFR2 or β-actin were purchased from Santa Cruz Biotechnology. Primary antibodies against p-ERK1/2 and p-Akt were purchased from Cell Signaling. ERK1/2 antibody and Akt antibody were purchased from Proteintech. Protein bands were developed using enhanced chemiluminescence on films or by fluorescence imaging.

### Tube Formation Assay and Cell Migration Assay

For tube formation assay, 1×10^4^ HUVEC cells were seeded in 96 well plates that were pre-coated with 50 µL/well Matrigel (BD Biosciences) and incubated at 37°C. About 16 hours later, capillary tubes formed were evaluated in random fields. For migration assay, intervened HUVEC cells were added to the upper chamber of 8.0-mm pore size 24-well transwell plates (Millipore). The lower chamber was filled with complete growth medium as a chemoattractant. After incubation at 37°C for 24 hours, the cells on the upper surface of the membrane were removed. The migrated cells were fixed and stained with 0.5% crystal violet buffer for about 20 minutes. The counted values of random fields under a microscope were statistically analyzed.

### Statistical Analysis

All data were presented as mean±SD and statistically analyzed using a Student’s *t* test. P value <0.05 was considered significant.
